# Automatic segmentation of myocardium at risk from contrast enhanced SSFP CMR: validation against expert readers and SPECT

**DOI:** 10.1186/1532-429X-18-S1-P222

**Published:** 2016-01-27

**Authors:** Jane Tufvesson, Marcus Carlsson, Anthony H Aletras, Henrik Engblom, Jean-Francois Deux, Sasha Koul, Peder Sörensson, John Pernow, Dan Atar, David Erlinge, Håkan Arheden, Einar Heiberg

**Affiliations:** 1 grid.4514.40000000109302361Dept. of Clinical Physiology, Skåne University Hospital in Lund, Lund University, Lund, Sweden; 2 grid.4514.40000000109302361Dept. of Biomedical Engineering, Faculty of Engineering, Lund University, Lund, Sweden; 3 grid.4793.90000000109457005Laboratory of Medical Informatics, School of Medicine, Aristotle University of Thessaloniki, Thessaloniki, Greece; 4 grid.412116.10000000122921474Dept. of Cardiology, Henri Mondor Hospital, Creteil, France; 5 grid.4514.40000000109302361Dept. of Cardiology, Lund University, Lund, Sweden; 6 grid.24381.3c0000000092415705Dept. of Medicine, Karolinska Institutet, Karolinska University Hospital, Stockholm, Sweden; 7 grid.55325.340000000403898485Dept. of Cardiology B, Oslo, University Hospital Ullevål and Faculty of Medicine, University of Oslo, Oslo, Norway

## Background

Efficacy of reperfusion therapy can be assessed as myocardial salvage index (MSI) by determining the size of myocardium at risk (MaR) and myocardial infarction (MI), (MSI=1-MI/MaR). Cardiovascular magnetic resonance (CMR) can be used to assess MI by late gadolinium enhancement (LGE) and MaR by either T2-weighted imaging or contrast enhanced SSFP (CE-SSFP). Automatic segmentation algorithms have been developed and validated for MI by LGE as well as for MaR by T2-weighted imaging. There are, however, no algorithms available for CE-SSFP. Therefore, the aim of this study was to develop and validate automatic segmentation of MaR in CE-SSFP.

## Methods

The automatic algorithm applies surface coil intensity correction and classifies myocardial intensities by Expectation Maximization to define a MaR region based on *a priori* regional criteria, and infarct region from LGE. Automatic segmentation was validated against manual delineation by expert readers in 183 patients with reperfused acute MI from two multi-center randomized clinical trials (RCT) (CHILL-MI and MITOCARE) and against myocardial perfusion SPECT in an additional set (n = 16). Endocardial and epicardial borders were manually delineated at end-diastole and end-systole. Manual delineation of MaR was used as reference and inter-observer variability was assessed for both manual delineation and automatic segmentation of MaR in a subset of patients (n = 15). MaR was expressed as percent of left ventricular mass (%LVM) and analyzed by bias (mean ± standard deviation). Regional agreement was analyzed by Dice Similarity Coefficient (DSC) (mean ± standard deviation).

## Results

**Figure 1:**
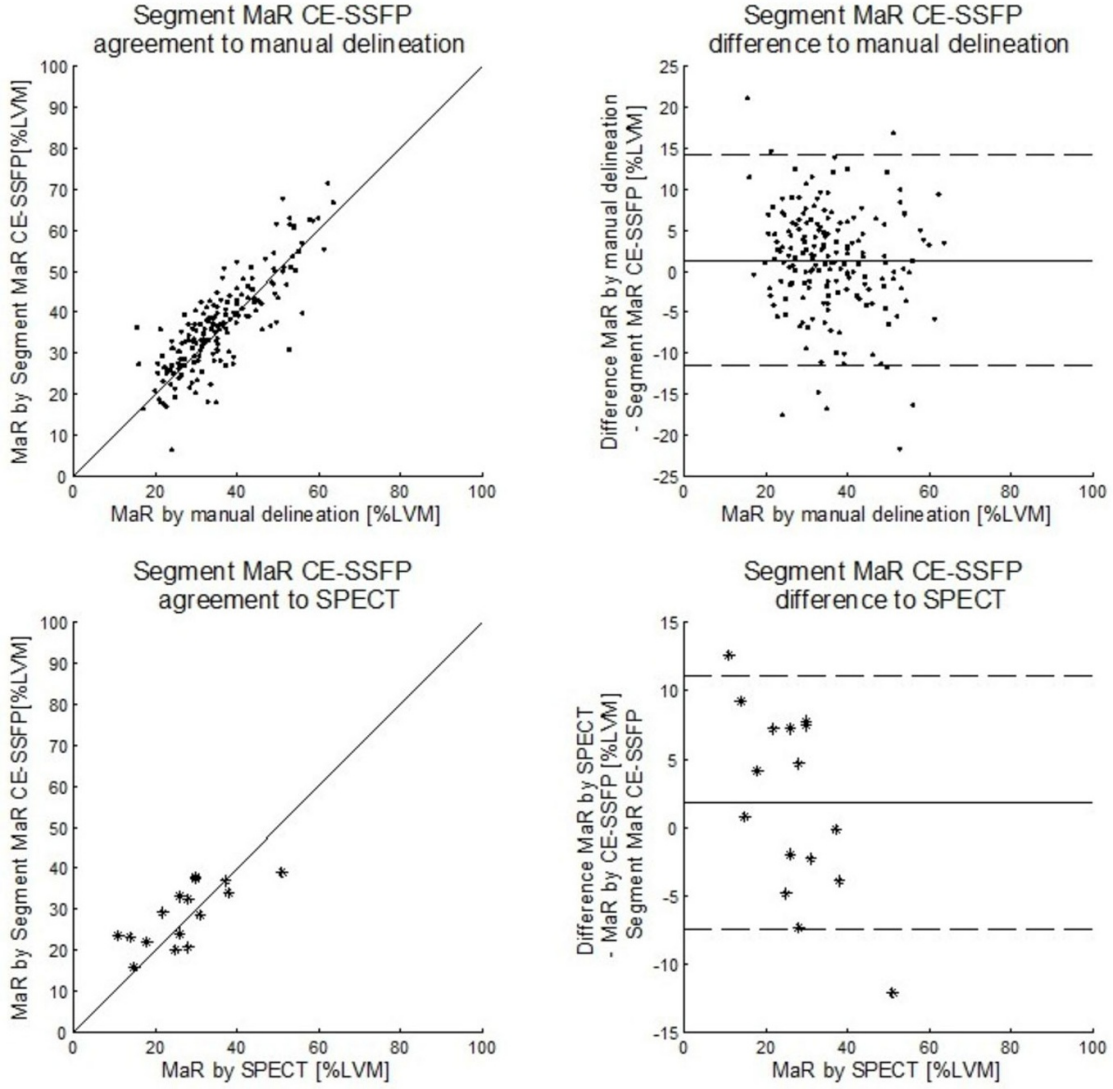
**Scatter plot of MaR as % of LVM (left column) and Bland-Altman plot of MaR bias as % of LVM (right column) for the automatic segmentation algorithm Segment MaR CE-SSFP against manual delineation in 183 patients (top row) and against SPECT in 16 patients (bottom row)**. The line of identity is shown as a solid line for both scatter plots and mean bias (solid line) and mean ± two standard deviations (dashed line) is shown for both Bland-Altman plots.

MaR assessed by manual and automatic segmentation were 36 ± 10 % and 37 ± 11 %LVM respectively with bias 1 ± 6 %LVM and regional agreement DSC 0.85 ± 0.08 (n = 183)(Figure 1). MaR assessed by SPECT and CE-SSFP automatic segmentation were 27 ± 10 %LVM and 29 ± 7 %LVM respectively with bias 2 ± 7 %LVM (Figure 1). Inter-observer variability was 0 ± 3 %LVM for manual delineation and -1 ± 2 %LVM for automatic segmentation.

## Conclusions

Automatic segmentation of MaR in CE-SSFP was validated against manual delineation in multi-center, multi-vendor studies with low bias and high regional agreement. Bias and variability was similar to inter-observer variability of manual delineation and inter-observer variability was decreased by automatic segmentation. Thus, the proposed automatic segmentation can be used to reduce subjectivity in quantification of MaR in RCT.

